# How and why party position estimates from manifestos, expert, and party elite surveys diverge: A comparative analysis of the ‘left–right’ and the ‘European integration’ dimensions

**DOI:** 10.1177/1354068821990298

**Published:** 2021-02-10

**Authors:** Alejandro Ecker, Marcelo Jenny, Wolfgang C Müller, Katrin Praprotnik

**Affiliations:** 26573 Universitat Mannheim, Germany; 27255 University of Innsbruck, Austria; 27258 Universität Wien, Austria; 31227 Donau-Universität Krems, Austria

**Keywords:** cross-validation, elite surveys, European integration, expert surveys, left–right party positions

## Abstract

This paper examines the validity of three approaches to estimate party positions on the general left–right and EU dimensions. We newly introduce party elite data from the comprehensive IntUne survey and cross-validate it with existing expert survey and manifesto data. The general left–right estimates generated by elites and experts show a higher congruence than those derived from party manifestos; neither measure clearly materializes as more valid regarding EU positions. We identify which factors explain diverging estimates. For instance, disagreement among experts has greater impact than their mere number. The substantial centrist bias of the manifesto estimates persists even when alternative documents are used to substitute manifestos. Low response rates among elites have no systematic detrimental effect on the validity of party position estimates.

## Introduction

Ever since Downs (1957) and Black (1958) disseminated the notion of spatial competition between political actors, scholars have been increasingly interested in estimating actors’ policy positions. In fact, locating the positions of political parties on a given policy continuum is an essential precondition for testing much of today’s theories of party competition, government formation, and legislative decision-making. For this purpose, political scientists have developed a variety of approaches for estimating the policy positions of political parties.

A direct consequence of this plurality of methods is a considerable controversy among scholars that revolves around the different methods’ validity and reliability. This is particularly true for the two most prevalent approaches for positioning political parties in a comparative framework: expert surveys and content analyses of party manifestos. Proponents of a document-based approach forcefully contest the validity of expert-driven data (Best, 2013; Budge, 2000, 2001; McDonald et al., 2007). Advocates of expert surveys, on the other hand, dispel the objections raised against the measurement quality and the inherent limitations of expert data (Hooghe et al., 2010; Steenbergen and Marks, 2007). Interestingly enough that despite the importance of the issue, only a few scholarly contributions have cross-validated the methods within one study in order to examine each measure’s relative validity (e.g. Bakker et al., 2015; Benoit and Laver, 2007; Hooghe et al., 2010; Keman, 2007; Marks et al., 2007; see also Adams et al., 2019 on party policy shifts). Moreover, looking at these few exceptions in greater detail (for an overview see Online Appendix Tables A.1.–A.3.), reveals that they only consider two methods at the same time or compare position estimates derived from different question wordings, especially due to the lack of comparable elite data – the third generally recognized source for party placements (Laver, 2014).

We aim to narrow this research gap and our analyses make the following three contributions to the field: First, we present a relevant addition to the field by cross-validating data from manifesto, expert, *and* elite studies that were not only generated at roughly the same time, but also relied on largely identical question wordings in the surveys at both a left–right and European integration dimension. This comprehensive approach is enabled by the IntUne elite survey, probably the largest survey ever conducted among political elites in Western Europe (Best et al., 2012). The IntUne data provide the rare opportunity to cross-validate party position estimates from expert and manifesto data using estimates derived from MPs as external yardstick. We argue that the IntUne data provide an adequate alternative take at party placements as long as the measurement error is not systematically correlated with both the expert and the manifesto data. This does not imply, however, that we see elite data as a gold standard of party position estimates. Rather, we adopt the logic of triangulation, which suggests that the amount of agreement of independent measures carries information on their validity (Marks, 2007). Our purpose thus is not to suggest using elite surveys instead of manifestos or experts for the spatial placement of political parties but to learn more about whether one of the two methods most frequently used in the context of the 21st century produces estimates that diverge from the placements derived from two equally credible sources.^1^ Second, we systematically discuss and test two sets of factors that help explaining given variances between elite data, on the one hand, and expert and manifesto data, on the other (i.e. manipulable measurement characteristics and party characteristics). Third, moving beyond simple cross-sectional analyses, we compare party position estimates not only on a general left–right dimension, but also on a European integration dimension on two points in time.

The logic of cross-validation of party position estimates rests on the assumptions i) that the different data sources all suffer from measurement error, and ii) that their systematic biases do not overlap much (Marks et al., 2007: 23). Accordingly, the standard approach to why measures diverge centres on the technical properties of sources and the process of extracting position data from them. While the present article places itself in this tradition it also asks in the concluding section whether the different nature of the sources affect the results and whether diverging party placements can be interpreted consistently once we consider categorial differences between the sources. If so, validity concerns need to be related also to the specific research questions to be addressed with the help of party position data.

## Expectations on differences between measures

Based on the literature on party position estimates, we develop our hypotheses on the differences between elite, expert, and manifesto data (please note that reviewing the entire spectrum of party position estimates is not our primary goal here, an overview can be found in the Online Appendix A as well as in previous studies such as Mair, 2001; Volkens, 2007). We provide a list of factors that might explain variation between party position estimates. In so doing, we group the factors into measurement characteristics and party characteristics. Our rationale is the following: It is reasonable to assume that the differences in the nature of the measures will result in differences in the party position estimates. However, these differences become problematic once they vary systematically with either the measurement or the party at hand. Systematic errors will lead to biased results in our analyses based on individual party position estimates.

### Manipulable measurement characteristics

Looking at the literature on party position estimates suggests that a number of characteristics of the measurement instrument drive measurement bias (e.g. Mair, 2001; Volkens, 2007). This is particularly interesting for scholars as they are, at least in principle, manipulable by the researcher. Specifically, the aggregate nature of the policy positions derived from expert surveys suggests that the validity of expert-based measures is a function of i) the number of experts evaluating the party positions and ii) the extent of disagreement among experts (Hooghe et al., 2010; Marks et al., 2007; Steenbergen and Marks, 2007). We thus expect increasing the number of experts to reduce systematic differences between measures. At the same time, higher levels of disagreement among experts should increase systematic differences between measures.

The same is true for position estimates derived from elite surveys. In fact, low response rates are one of the main critiques of surveys among political elites, in particular, as responsive MPs are unlikely to be a random sample of all party MPs and their opinions are therefore in some respect likely to be unrepresentative of the party as a whole. Recent research on two-party systems finds little empirical evidence that elite surveys with lower response rates are necessarily less representative of the population (Fisher and Herrick, 2013). Yet in multi-party systems extremist parties might be underrepresented and even respondents from such parties might be less radical than non-respondents. While we cannot address all potential concerns associated with elite data, below we explore empirically whether reducing the share of respondents adversely affects the correspondence between measures.

Concerning estimates derived from election manifestos, two particular characteristics of the party documents may drive systematic differences between measures. A first rather straightforward criterion is simply the total amount of textual data provided. Thus, succinct pamphlets will yield more imprecise measurements than lengthy accounts on a wide range of policy issues (Marks et al., 2007: 27). A second aspect that has attracted considerable attention among critiques of the MARPOR/CMP project is document selection (Gemenis, 2012, 2013; Hansen, 2008).^2^ The Party position estimates are quite often derived from general party programs (e.g. *Grundsatzprogramme* for the case of Germany) that are not directly linked to a particular election, from joint electoral manifestos of parties forming pre-electoral alliances, or from speeches of leading candidates during the election campaign. If neither of these alternative sources for party-specific election manifestos is available, party position estimates are simply obtained via interpolation. Naturally, document selection may thus be a key factor in explaining systematic differences between measures.

### Party characteristics beyond control of researchers

Beyond these measurement attributes, party characteristics out of the researchers’ control may likewise contribute to systematic differences between measures. One such factor is party extremism that scholars hypothesize to have detrimental effects on estimates derived from party manifestos. Assuming that parties reveal their ‘true’ positions in their manifestos, coder misclassification of coding units will have a disproportionate effect on ‘extreme’ parties as coders can increasingly err towards the centre the closer parties are to either extreme of a given scale. As a result, these parties are likely to be coded more moderate than their ‘true’ policy position, which leads to a centrist bias of position estimates derived from party manifestos (Mikhaylov et al., 2012). If survey recruitment from extremist parties is indeed biased towards the more moderate MPs, the same centrist bias should affect measures from elite surveys.

Concerning expert estimates, several authors concur that their ability to pinpoint parties’ ‘true’ policy positions is positively associated with party extremism (Hooghe et al., 2010; Marks et al., 2007; Netjes and Binnema, 2007). A stronger differentiation from other parties presumably facilitates the acquisition of information and thus reduces the uncertainty of experts where to place these parties on a given policy continuum. In contrast, Lindstädt et al. (2020) suggest that experts assessing parties at the extremes of the scale can, similar to manifesto coders, only make mistakes towards the middle of the scale. Overall, the expectations on the effect of party extremism on expert estimates are thus conflicting.

Furthermore, higher levels of intra-party divergence are expected to severely obstruct experts’ ability to evaluate parties. Given that the party leadership utters highly conflicting policy positions, the resulting cacophony will render it virtually impossible, even for experts, to discern a single coherent policy position. Party manifestos are equally assumed unable to accommodate divergence within political parties. Accordingly, manifestos of internally divided parties are likely to yield more imprecise position estimates (Marks et al., 2007: 27). Intra-party divergence will also affect estimates derived from elite surveys. In fact, the validity of the aggregate party estimate derived from MPs individual response will naturally decrease as the extent of ideological conflict within political parties increases.

An additional factor that restrains experts’ ability to unambiguously identify parties’ policy positions are substantial policy shifts (Marks et al., 2007: 27). In line with the literature on voters’ perceptions of policy positions (e.g., Dahlberg, 2009), experts are similarly expected to have a hard time determining parties’ policy positions when faced with a highly erratic party system. Since manifesto-based estimates are based upon one single document for each time point, the measurement is unaffected by policy shifts and this factor is therefore excluded from the analysis. The same holds true for estimates derived from political elite surveys.

### Additional controls

Following Marks et al. (2007: 27–28), we also control for another set of potential explanatory factors for differences between elite data, expert judgments, and manifesto estimates. The first is party size, as all data should yield results that are more congruent as party vote share increases. Large parties are present in the experts’ minds and it seems reasonable to expect that experts thus hold more precise information concerning the policy positions of larger parties. Larger parties should also provide more survey respondents. With regard to party manifestos, Gabel and Huber (2000) conjecture that parties with a large electoral basis are more likely to provide informative manifestos, while smaller parties may cater to a particular target electorate and thus emphasize specific policy issues. This, in turn, may jeopardize the validity of position estimates based on smaller parties’ manifestos.

A similar argument holds true for party age. Again, it seems plausible to expect that long-established parties are not only actors visible and well known to the experts but that older and established parties also publish manifestos that are more thorough and provide internal and external orientation. Both effects should thus decrease any systematic differences between measures. Another potentially relevant factor for the validity of party position estimates beyond party size and age is government status. Here, we expect government parties to thoroughly instruct their MPs and to be more visible to experts since these actors are largely responsible for current policymaking. Opposition parties may emphasize specific policy dimensions in order to highlight the government’s most startling deficiencies. Incumbent government parties, on the contrary, will address a broader range of issues as they vindicate their decisions during their term in office. The electoral manifestos of opposition parties may thus be less suited to extract party stances than those of government parties.

We also expect that the issue dimension under scrutiny affects the differences between party position estimates. Here, we hypothesize that an increased relevance of the policy dimension at hand will decrease the differences between elite and both expert and manifesto data (Hooghe et al., 2010; Marks et al., 2007; Netjes and Binnema, 2007). A final factor for systematic measurement error by experts, loosely related to the stability and experts’ familiarity with of the party system, is whether the parties are from Eastern or Western Europe. Both the empirical findings by Benoit and Laver (2007) and Hooghe et al. (2010) suggest that the policy stances of Eastern European parties are more difficult to grasp for experts.^3^ Frequent switching of MPs between parties in these countries (Tavits, 2013) suggests a similar effect at the elite level. We will therefore include that factor with regard to expert data. Table 1 summarizes our expectations on the systematic differences between party position estimates.

**Table 1.** Expected differences between expert- and manifesto-based measures.

	Experts	Manifestos
**Manipulable characteristics**		
Number of experts	–	
Expert agreement	–	
Per cent of MPs	–	–
Type of document (party-specific)		–
Length of document		–
**Characteristics beyond control**		
Intra-party heterogeneity	+	+
Party position shift	+	
Party extremism	+/–	+
**Controls**		
Party size	–	–
Government party	–	–
Party age	–	
Issue salience	–	–
Central and Eastern Europe	+	

*Notes:* (+) denotes factors that increase systematic differences between measures, (–) denotes factors that decrease systematic differences between measures; () not applicable.

## Data and methods

Our empirical analysis for cross-validating the party position estimates is based on a *quadripartite* data structure. The elite data on both dimensions are retrieved from the IntUne elite surveys administered in early 2007 and late 2009 to about 1,400 MPs and 1,100 MPs, respectively, in both Western and Central Eastern European countries (Cotta et al., 2007, 2009). This rich data set features, inter alia, individual-level data on the respondents’ self-placement on a general left–right scale, their attitude towards the European integration project, alongside their party affiliation. Most importantly for our purposes, the question wording on the policy positions in both dimensions is very similar to that used in the CHES expert surveys.^4^ Based on these individual-level data we obtain mean position estimates of 217 political parties in 17 European Union (EU) member states.

The estimates of country experts are obtained from the 2006 and the 2010 CHES data sets. The 2006 survey was completed by 235 country experts providing data on the left–right positions as well as the stance on European integration of 227 parties in 22 EU member states (Hooghe et al., 2010). Analogously, the 2010 CHES data set contains the policy positions of the party leadership of 238 parties in 28 countries (Bakker et al., 2015).

The MARPOR/CMP data is the most prominent and comprehensive source for manually coded party position estimates.^5^ It comprises manifesto-based estimates on the general left–right dimension (Volkens et al., 2015). More precisely, each party’s overall ideological stance is derived from the manifesto published in the context of the most recent national election prior to 2006 and 2010, respectively. Given that political parties frequently shift their policy positions (Adams, 2012; Laver, 2005), this approach mitigates the problem that the absolute difference between measures may be a simple artefact of party policy shifts over time.

The EES data set finally includes estimates on the European integration dimension, based on Euromanifestos for the European Parliament elections in 2004 and 2009 (Braun et al., 2015).^6^ The data set contains 783 manifestos of 289 different parties in 27 countries and of the European Parliament groups. Overall, combining these four different data sources into a single coherent data set leaves us with a pooled cross-national sample covering 131 political parties in 17 EU member states (see Online Appendix Table A.4. for additional information on the quadripartite data structure).

Naturally, a first prerequisite of analysing differences between elite, expert, and manifesto scores is to extract single party policy positions.^7^ As indicated above, we use simple averaged individual evaluations in order to retrieve the position estimates of elites and experts on both the left–right and the European integration dimensions. These measures have been used in earlier cross-validation research and thus facilitate comparison. While both elites and experts assess each party’s overall ideological orientation on an 11-point left–right continuum, the IntUne and the CHES surveys apply different scales for capturing positions on European integration. Specifically, MPs use an 11-point scale while country experts use an alternative scale running from 1 to 7. We thus opt to rescale these position estimates on a scale ranging from 0 to 100.

Concerning the MARPOR/CMP data there are a series of rival approaches to retrieve position estimates on both the left–right and the European integration dimensions. Here, we opt for the logit scale developed by Lowe et al. (2011), which has materialised as the de-facto standard in research on party policy positions. Specifically, party *i’s* left–right position is given by the (logged) total number of right statements, relative to the total number of left statements in its party document


$$Leftright_{i}=\text{log}\frac{right\;statements_{i}+0.5}{left\;statements_{i}+0.5}$$

In a similar vein, the estimates on European integration in the EES data are likewise operationalized using a logit scale. Party *i’s* position on the European integration process is defined as the logged ratio of positive versus negative statements on European integration


$$European\;integration_{i}=\text{log}\frac{pro\;EU_{i}+0.5}{anti\;EU_{i}+0.5}$$

Unlike various alternative scaling approaches such as the ratio scale proposed by Kim and Fording (1998, 2003), the logit scale does not make any assumptions about its endpoints. To ensure comparability across data sources, we rescale the left–right estimates derived from the MARPOR/CMP data and the EU position estimates based on the EES data likewise from 0 to 100 based on the *observed empirical* endpoints of the scale.^8^

Capturing most of the measurement and party characteristics discussed in the previous section is rather straightforward. Here, we focus on the operationalization of determining factors of substantial interest, while we discuss the measurement of the control variables in more detail in the Online Appendix. The extent of intra-party heterogeneity is captured via the index of agreement originally proposed by van der Eijk (2001). This measure is particularly well suited for the analysis of ordered rating scales as it allows differentiating between the actual dispersion and the skewness of a distribution. In contrast to empirical measures resorting to the standard deviation it decomposes the frequency distribution into constituent layers, i.e. simple component parts for which agreement can be unambiguously defined. Given that our subject matter is intra-party divergence we reverse van der Eijk’s original scale. The hypothetical scenario in which half of a party’s MPs place themselves on each extreme of the ordered scale constitutes the measure’s upper bound (+1). The lower bound (−1) accordingly describes a situation in which all party elites place themselves in only one category, while a hypothetical uniform distribution yields an intra-party divergence score of 0. Similar to intra-party dissent, disagreement among experts is captured via the index of agreement.

Our measures of party policy shifts in each dimension are based upon the MARPOR/CMP data set. In line with the vast majority of the literature on the dynamics of party positions (Adams, 2012) we operationalize policy shifts as the absolute difference between a party’s policy position at *t*, i.e. in the context of the most recent election prior to 2006 and 2010, and the same party’s position at *t* − 1, i.e. the next to last election. For those parties for which only a single position at *t* could be retrieved – mostly due to parties being founded only after *t* − 1 – we assume perfect stability of these parties’ policy positions.

Finally, we largely follow Dahlberg’s approach (2009: 274) to capture the level of extremism of a party’s policy position. In this context, extremism is defined as the weighted average policy distance of a party to all other parties in a country’s party system with the weights being the other parties’ vote share. The major advantage of this measure is that policy distances from smaller extremist parties do not exert a disproportionate effect on the overall measure. Formally, the extremism of party *i* is defined as


$$extremism_{i}=\frac{\sum_{j\neq i}v_{j}|p_{i}-p_{j}|}{\sum_{j\neq i}v_{j}}$$

where *v_j_* is the vote share of party *j* and *p_i_* and *p_j_* are the policy positions of party *i* and *j* respectively as derived from the IntUne elite survey.

## Cross-validation of elite, expert and manifesto data

A crucial precondition for assessing each measure’s relative validity is to establish that all three measures relate to the same latent concept. Hence, we start our empirical section with two exploratory principal axis factor analyses before delving into the analysis of contributing factors to systematic differences between measures. The rationale for this analysis is twofold: first, it allows examining whether it results in a single factor solution suggesting that all three measurements load on a single, common factor (Marks, 2007). Second, the factor analysis allows us to capitalize on the unique and comprehensive information provided by the IntUne data. In fact, the rationale of triangulation suggests that the systematic error component differs across measures of policy positions. Therefore, triangulating the two conventional measures based on manifesto and expert data with position estimates derived from hard to obtain elite data results in a combined measure with substantially reduced systematic error which closer approximate parties’ ‘true’ policy positions (Marks et al., 2007: 25). Consequently, we are able to show which of the regularly generated and updated party position estimates – expert or manifesto data – load more heavily on the common factor and thus, provide more valid information.

The first column in Table 2 presents the results of an exploratory principal axis factor analysis of the left–right policy positions derived from elite, expert, and manifesto data. Using the general eigenvalue cut-off point of 1.0 gives us a single factor solution. As apparent from the scheme, not only elite data, but also expert and manifesto data exhibit high factor loadings, indicating that these measures share a substantial amount of variance. At the same time, the position estimates retained from country experts load heavily on the common factor while the manifesto measure yields a considerably lower factor loading. Thus, according to the rationale of triangulation, experts seem to be the more valid data source of left–right position estimates.

**Table 2.** Exploratory factor analysis and concordance correlation of left–right estimates.

	Factor loading	Elite data		
		*ρ_c_*	*C_b_*	*ρ*
Elite data	0.92			
Expert data	0.92	0.87	0.99	0.88
Manifesto data	0.67	0.36	0.58	0.63

*Notes:* N = 128.

*Sources:* Bakker et al. (2015), Cotta et al. (2007, 2009), Hooghe et al. (2010), and Volkens et al. (2015).

A similar pattern results from exploring the bivariate relationship between the estimates derived from the IntUne elite survey and the policy positions generated by the CHES expert survey and those based on party manifestos (for a similar approach, see e.g. Gemenis, 2012). Here, the rationale is using elite data largely as external benchmark, which allows assessing the relative validity of expert and manifesto data vis-à-vis a third self-contained data source. Specifically, columns 3 to 5 in Table 2 show the concordance correlation between elite data and expert and manifesto estimates. The concordance correlation coefficient evaluates the agreement between measurements, takes both systematic differences (or accuracy denoted by *C_b_*, i.e. does the best-fit line approach the line of perfect concordance) and random measurement error (or precision denoted by *ρ*, i.e. do the data approach that best-fit line) into account, and allows differentiating between the two (Lin, 1989, 2000).^9^ In sum, the positive concordance correlation denoting overall agreement suggest that the relationship between elite data and both expert measures and manifesto estimates is considerably strong. However, the concordance between elite and expert data (*ρ_c_* = 0.87) is substantially higher than that between elite and manifesto data (*ρ_c_* = 0.36). Most interestingly, we observe that most of the disagreement between elite and expert data is due to random error (indicated by the very high *C_b_*), while the higher disagreement between elite and manifesto data is at least partly attributable to an increase in systematic differences (denoted by the decrease in *C_b_*).

**Figure 1.**
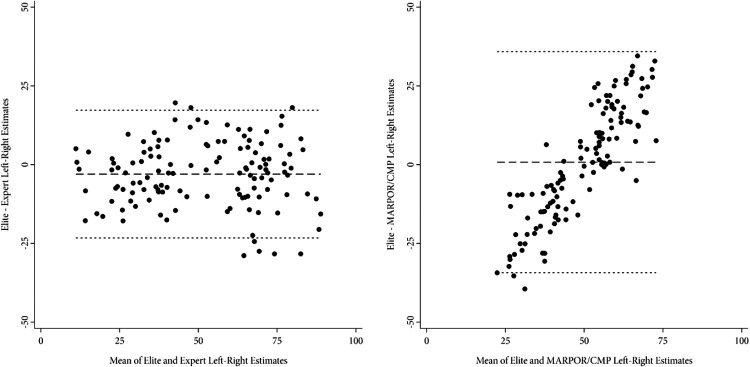
Bland-Altman Plot of left–right estimates. *Sources:* Bakker et al. (2015), Cotta et al. (2007, 2009), Hooghe et al. (2010), and Volkens et al. (2015).

The Bland-Altman plot depicted in Figure 1 corroborates this notion. The left panel juxtaposes the difference between elite and expert left–right positions estimates against the mean of the two measures. The right panel plots differences between left–right estimates derived from elite and the MARPOR/CMP data against the mean thereof. These Bland-Altman plots allow exploring and detecting differences between measures due to systematic bias.^10^ As such, the two strikingly different patterns in Figure 1 provide considerable support for the initial assertion. Specifically, the line of observed average agreement in the left panel approximates zero, while the observations cluster around this observed average agreement and their distribution largely follows a random pattern. In contrast, the observations in the right panel seem more dispersed and show a considerable positive linear relationship. This, in turn, implies a systematic relationship between party stance and measurement error.

Specifically, the right panel displays the characteristic *centrist bias* often found when exploring the validity of document-based approaches to left–right position estimates (e.g. Benoit et al., 2012; Gemenis, 2012). The difference between measures is thus particularly large for political parties at either end of the political spectrum, which the MARPOR/CMP estimates consistently identify as more moderate than the estimates derived from the IntUne elite data. For moderate parties in the centre of the ideological distribution, in contrast, Figure 1 indicates high agreement between estimates derived from elite and MARPOR/CMP data.

Turning to the position estimates on European integration, a somewhat different empirical picture emerges. The results of the exploratory factor analysis in Table 3 indicate that the three measures feature on average slightly lower factor loadings. Thus, policy positions on European integration retrieved from elite, expert, and Euromanifesto data are somewhat more difficult to array into a single underlying dimension. Most strikingly, however, the results in Table 3 indicate that elite, expert, and Euromanifesto data exhibit similar factor loadings. According to the rationale of triangulation, thus neither of these measures has a considerable competitive advantage as the most valid data source for estimating stances on European integration.

**Table 3.** Exploratory factor analysis and concordance correlation of EU estimates.

	Factor loading	Elite data		
		*ρ_c_*	*C_b_*	*ρ*
Elite data	0.76			
Expert data	0.79	0.60	0.91	0.66
Manifesto data	0.71	0.56	0.97	0.58

*Notes:* N = 110.

*Sources:* Bakker et al. (2015), Cotta et al. (2007, 2009), Hooghe et al. (2010), and Braun et al. (2015).

Exploring the bivariate relationships between measures corroborates this finding. Again, the concordance correlation coefficients in Table 3 indicate that all three measures are positively and significantly interrelated. Yet, compared to the left–right position estimates the level of agreement between elite and expert data seems considerably smaller. As a result, the concordance between elite and expert data (*ρ_c_* = 0.60) is now largely comparable to that between elite and Euromanifesto data (*ρ_c_* = 0.56).^11^ At the same time, Table 3 likewise indicates that this decrease in convergence is driven by both an increase in the systematic and the random error components (as indicated by the decrease in *C_b_* and *ρ*. Part of this weaker interrelation between measures for the elites–experts dyad – compared to the left–right estimates – may be partly due to the different scales applied to derive EU position estimates (7-point scale for expert evaluations and 11-point scale for elite surveys).

**Figure 2.**
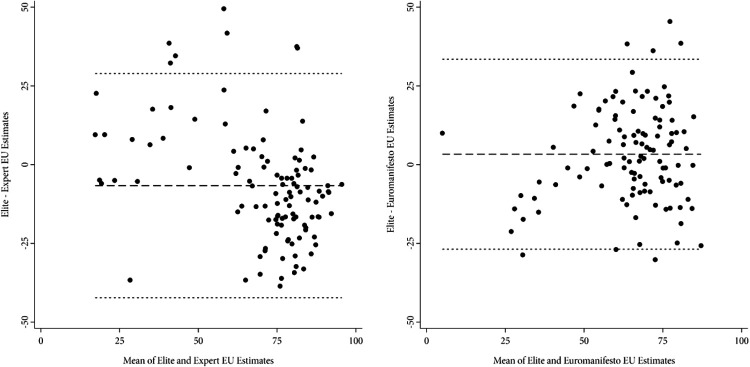
Bland-Altman plot of EU estimates. *Sources:* Bakker et al. (2015), Cotta et al. (2007, 2009), Hooghe et al. (2010), Braun et al. (2015).

Plotting the difference between European integration against the mean of these estimates shows two characteristic patterns for both the elites–experts and the elites–Euromanifesto dyads (see Figure 2). First, agreement between measures is particularly low for parties that generally support the European integration process. Specifically, both expert and Euromanifesto often indicate very strong support for European integration among parties that based on estimates derived from elite data are merely moderately supportive of EU integration. Second and related, within this large cluster of supportive parties, agreement between measures is even lower as elite data reveal subtle but important differences among pro-European parties that both expert and Euromanifesto data are unable to uncover (Proksch and Lo, 2012a; see also Marks et al., 2012 and Proksch and Lo, 2012b).

## Examining systematic differences between measures

Do we find any systematic patterns that account for the extent to which expert placements and estimates derived from party manifestos differ from the self-placements of political elites? We explore potential differences by first regressing the elite estimates on the expert and (Euro) manifesto estimates respectively^12^ and then use the absolute residuals as dependent variables to explore potential systematic differences. The rationale for this approach is to obtain predictions of party placements based on expert placements and party manifestos and to exploit the (absolute) residuals to capture the extent to which these predictions deviate from the observed elite estimates (Marks et al., 2007: 28). Put differently, the residuals should display the characteristic white noise pattern if there were no systematic differences between the self-placements of political elites and expert placements and estimates derived from party manifestos, respectively. Table 4 displays the results of the four corresponding ordinary least squares regression models with heteroscedasticity robust standard errors.^13^

The first model examines systematic differences between left–right policy positions derived from experts and elites. As corroborated, we observe that systematic differences between position estimates decrease as agreement among experts increases. Specifically, shifting from no agreement (experts randomly placing parties resulting in a uniform distribution) to perfect agreement (all experts placing a party on the same position of the ideological spectrum) reduces the difference between measures by approximately eight scale points (range 0 to 100). This corresponds to 1.5 times the root-mean-square error. In contrast, policy positions shifts reduce systematic differences between experts and elites. Thus, contrasting the conventional empirical picture, experts seem well aware of party position shifts and in fact, these shifts seem to encourage expert observers of party competition to ‘update’ their assessment of parties’ ideological position, which in turn reduces systematic differences. Finally, we observe that increasing the share of responding MPs adds to the systematic differences between left–right policy positions derived from experts and elites, suggesting that the number of elite respondents might be more important in densely populated party systems than recent work on two-party systems suggests.

**Table 4.** Explaining differences between measures.

	Left–right		European integration	
	Experts	Manifestos	Experts	Euromanifestos
	(1)	(2)	(3)	(4)
**Manipulable characteristics**				
Number of experts	0.04 (0.11)		−0.23 (0.17)	
Expert agreement	−8.16* (3.91)		−9.90* (4.86)	
Per cent of MPs	0.08*** (0.02)	0.03 (0.04)	0.00 (0.04)	0.01 (0.05)
Type of document				
Single party	*Reference group*		*Reference group*	
Two or more parties		1.58 (5.08)		
Estimate		12.42*** (2.32)		
Main party		3.86 (3.46)		
Party bloc		−9.63*** (2.85)		
Party leader				3.44 (2.91)
National manifesto				3.11 (5.64)
Other programme		−2.30 (2.50)		0.16 (2.16)
Length of document		−1.01 (0.86)		1.27 (0.78)
**Characteristics beyond control**				
Intra-party heterogeneity	6.73 (5.46)	4.62 (8.73)	15.01** (5.56)	6.44 (5.11)
Party position shift	−0.11** (0.03)		−0.06 (0.26)	
Party extremism	−0.36 (0.57)	4.77*** (0.81)	1.42* (0.71)	4.40*** (0.79)
**Controls**				
Party size	0.09 (0.05)	−0.01 (0.08)	−0.06 (0.07)	−0.18 ** (0.07)
Government party	−0.84 (0.92)	0.43 (1.72)	0.01 (1.57)	−0.03 (1.45)
Party age	−0.03 (0.03)	−0.07 (0.05)	−0.02 (0.04)	−0.02 (0.04)
Issue salience	5.23 (5.74)		−1.20 (1.55)	
Central and Eastern Europe	3.41** (1.28)	−1.09 (2.22)	−1.74 (1.98)	−1.40 (1.95)
Constant	3.58 (6.88)	4.11 (8.68)	14.27* (6.53)	−2.58 (6.88)
R^2^	0.29	0.30	0.27	0.39
Observations	123	129	123	109

*Notes:* Robust standard errors in parentheses, * p < 0.05, ** p < 0.01, *** p < 0.001.

*Sources:* Bakker et al. (2015), Cotta et al. (2007, 2009), Hooghe et al. (2010), Volkens et al. (2015), Braun et al. (2015).

The second model juxtaposes the left–right estimates generated by MARPOR/CMP with those estimates derived from political elites. As hypothesized, the selection of coded documents has a substantial effect on the systematic differences between estimates. However, it is the *estimated* or *interpolated* estimates rather than those derived from party bloc programmes or other documents such as election speeches that disproportionately contribute to differences between measures. In line with the bivariate picture drawn in Figure 1, the multivariate analysis in Table 4 likewise indicates a considerable centrist bias of estimates based on election manifestos. Political parties located at either end of the left–right ideological spectrum show significant and substantial differences as increasing party extremism by one standard deviation increases the difference between measures by approximately 4.8 scale points. This, in turn, corresponds to half the root-mean-square error.

Examining systematic differences between expert and elite estimates on European integration (model 3), further corroborates our initial empirical findings. As with the left–right estimates, we observe that aggregating highly conflicting expert responses on party positions regarding European integration leads to increased systematic differences between measures. In contrast, higher agreement among political elites increases systematic bias. Although small and homogenous regionalist parties, which portray themselves as unanimously pro EU, drive this finding, it indicates that intra-party divergence has at least no systematic *negative* effect on European integration estimates derived from political experts.

The fourth and final model examines systematic differences between European integration position estimates derived from elites and estimates derived from Euromanifestos. In contrast to the position estimates on the left–right ideological continuum, we observe that document selection has no influence on systematic differences between measures. In this context, however, it is worth noting that over 90% of the position estimates are derived from ‘proper’ Euromanifestos and hence document selection is generally less problematic. In addition, Table 4 indicates that parties with more extreme positions on the European integration dimension display disproportionate differences between both sets of estimates. This seems to contradict the bivariate assessment of ‘moderate’ parties driving differences between measures. In this context, however, even moderately pro-European parties qualify to a certain extent as ‘extremist’ parties as the operationalization is based on the empirical distribution rather than the potential range of values on the European integration dimension. Finally, the elite–Euromanifestos dyad is the only one, where we observe decreasing differences between measures with increasing party size.

While several of our findings pertain to specific measures or a particular dimension of party competition, the empirical analysis results in a number of general findings. Some of these confirm established empirical patterns. At the same time, however, the analysis also refines several of these established findings and adds new insights into the relative strengths and weakness of established measures. Frist, contrasting much of the conventional wisdom in the literature, we find no systematic effect of the number of experts on measurement differences. Thus, rather than low response rates, it is disagreement among experts that appears to be a key challenge for these estimates. This, in turn, has substantial implications for the quality of aggregated expert responses (Lindstädt et al., 2020). Second, intra-party heterogeneity has no effect on systematic differences between measures for the elites–experts and the elites–(Euro) manifesto dyads, respectively. Thus, neither measure enjoys a competitive advantage in dealing with conflicting policy positions within parties (this is not to say that intra-party heterogeneity affects all measures equally). Third, the selection of documents is one of the main challenges for document-based approaches interested in left–right position estimates (Gemenis, 2012). Yet, the substantial centrist bias of the MARPOR/CMP estimates persists beyond the use of alternative documents, and is thus only partly attributable to the higher share of non-coded coding units (i.e. quasi-sentences) in the various alternative documents. At the same time, we do not find document length to affect adversely the estimates derived from electoral manifestos. Finally, our findings confirm that the generally low response rates among political elites have no systematic detrimental effect on the validity of party position estimates (Fisher and Herrick, 2013).

## Discussion

This article presents a further step in disentangling the variances between party position estimates derived from elite, expert, and manifesto data. In so doing, our study moves beyond existing research in three important aspects: First, we test two sets of factors that explain observed differences between elite data, on the one hand, and expert and manifesto data, on the other. Second, we move beyond simple cross-sectional analyses and compare party position estimates not only on a general left–right dimension, but also on the European integration dimension. Third, our study benefits from the fact that all measures coming from four different data sources are generated almost at the same point in time. It seems thus very unlikely that our results are biased due to simple party switches or changes in party positions over time.

The empirical analysis results in two key findings: First, the general left–right estimates generated by elites and experts show a higher congruence than those derived from party manifestos. Concerning European integration, in contrast to the earlier findings of Marks et al. (2007), neither measure clearly materializes as more valid approximation of party positions. This again highlights that source selection – in our case Euromanifestos instead of national manifestos – and scaling approach – log-ratio scale instead of conventional ratio and difference scaling – are consequential choices. Second, several factors account for systematic differences between estimates derived from elite surveys and those derived from the other two sources. We find disagreement among experts substantially more important than the mere number of experts. Neither the measure based on elites nor experts enjoys a competitive advantage in dealing with conflicting policy positions within parties. We confirm the substantial centrist bias of the MARPOR/CMP estimates that persists even when alternative documents are used. Finally, our findings confirm that the generally low response rates among political elites have no systematic detrimental effect on the validity of party position estimates.

In introducing the three sources from which party positions are derived, we have hinted at differences between them due to their different nature. Specifically, they can be seen as being more or less strategic. Party manifestos are written to convey the image that parties consider electorally advantageous. Yet in so doing, parties are constrained by their records, activists, and credibility considerations. Still, the general take at party self-positioning is that it has to be taken with a grain of salt, that there is a need for ‘discounting’ to arrive at the parties’ ‘true’ positions. Party elites, answering spontaneously, anonymously, and without immediate electoral context, should be less strategic. Independent experts, in turn, should be non-strategic. The more important a policy dimension is, the more strategic party self-placement should be and the more estimates derived from these different sources should diverge. Left–right has been the dominant conflict dimension at the time of investigation and here we find divergence between our sources corresponding with these considerations as the party positions derived from the most strategic source are the most centrist while those from the lest strategic source, are the most extreme.

Our results can help researchers to make better-informed choices between party position estimates when seeking to explain substantive phenomena with the help of existing party placement data. Generally, cross-validation suggests that expert data provide more valid estimates on the left–right dimension while expert and manifesto data seem equally trustworthy with regard to the European integration dimension. If we relate to specific research questions, however, and consider the nature of the data sources, more nuanced considerations come into play. For instance, when researchers are interested in parties’ campaign behaviour in the run-up to an election, manifestos provide unique and comprehensive insights into parties’ strategic positions. Questions that refer to parties’ actual behaviour during the legislative period, on the other hand, might be better answered based on estimates from expert surveys unless there are strong reasons to believe that the specific party behaviour has had a major impact on how the experts perceive party placements.

## Supplemental material

Supplemental Material, sj-docx-1-ppq-10.1177_1354068821990298 - How and why party position estimates from manifestos, expert, and party elite surveys diverge. A comparative analysis of the ‘left–right’ and the ‘European integration’ dimensions Click here for additional data file. Supplemental Material, sj-docx-1-ppq-10.1177_1354068821990298 for How and why party position estimates from manifestos, expert, and party elite surveys diverge. A comparative analysis of the ‘left–right’ and the ‘European integration’ dimensions by Alejandro Ecker, Marcelo Jenny, Wolfgang C Müller and Katrin Praprotnik in Party Politics

## Notes

1. The only cross-validation including elite data is confined to the European integration dimension in the context of the 1990s (Marks et al., 2007).

2. Although political parties naturally determine type and length of their election manifesto, we argue that researchers have – at least to some extent – discretion over these characteristics. Specifically, obtaining the ‘right’ party documents often requires substantial archival work. In a similar vein, researchers may choose to complement shorter election manifestos with additional textual data derived from leader speeches, for instance.

3. Specifically, Rohrschneider and Whitefield (2016) suggest that Eastern European parties have accumulated less reputation for their positions and thus are more flexible in changing them. This, in turn, makes them moving targets, which are harder to pin down for experts.

4. See the Online Appendix for the exact question wording.

5. There is, however, a vivid and ongoing debate about the validity and reliability of the data (e.g. most recently discussed in Budge et al., 2013; Budge and Meyer, 2013; Gemenis, 2012, 2013). Our analyses help to systematically analyse the respective claims by cross-validating the MARPOR results with the IntUne data.

6. Although, as mentioned above, some estimates are derived from speeches of leading candidates rather than from party-(group)-specific election Euromanifestos. Note that all substantive conclusions about the different measures’ relative validity are robust to using position estimates on European integration derived from national party manifestos (Volkens et al., 2015).

7. We are aware of various methods designed to estimate more valid estimates of party positions from one source of data (e.g., Bakker et al., 2014; König et al., 2013) or across data sources (e.g. Lo et al., 2014). However, we abstain from using these methods as each of them may improve the quality of one or some estimates but not all. While we would recommend the use of such methods in substantive applications of the data, they might bias our comparisons over different types of data.

8. All key findings are robust to using an alternative ratio scale employed in the cross-validation analyses by Kim and Fording (2002), Marks et al. (2007), and Ray (2007) with an explicit range constraint from –1 to 1.

9. For an in-depth discussion on the difference between agreement and linear relationship between measures, see, for instance, Altman and Bland (1983) and Bland and Altman (1999).

10. Similar to residual versus fitted plots.

11. Note that this is an important difference to the finding reported in Marks et al (2007), which, however, is based on a different data source (national vs European manifestos) and a different scaling approach.

12. An alternative approach would be regressing the factor scores of the above factor analysis on the expert and (Euro) manifesto estimates and then use these residuals as dependent variable. Here, however, information from expert and (Euro) manifesto estimates would affect both sides of the equation and potentially blur systematic differences between measures.

13. See Table A.5. in the Online Appendix for a quick overview of the key determinant factors.
