# Supplementary material for: How and why party position estimates from manifestos, expert, and party elite surveys diverge: A comparative analysis of the ‘left–right’ and the ‘European integration’ dimensions
Source: Party Politics. 2021 Feb 10;28(3):528–40. doi: 10.1177/1354068821990298 (PMC9036146; doi:10.1177/1354068821990298)
Supplement: Supplemental Material, sj-docx-1-ppq-10.1177_1354068821990298 - How and why party position estimates from manifestos, expert, and party elite surveys diverge. A comparative analysis of the ‘left–right’ and the ‘European integration’ dimensions [file sj-docx-1-ppq-10.1177_1354068821990298.docx]

# Online Appendix

**A. Information on party position measurements**

Party position estimates are a *sine qua non* for solving research puzzles in a variety of sub-fields such as coalition politics or electoral competition. In this paper, we cross-validate party position estimates from three different data sources: elites (IntUne data), experts (CHES data), and party manifestos (MARPOR/CMP data; please see Online Appendix C for detailed information on the data sets). Our results should help researchers to select the adequate data source for answering their research question or to better understand the implications of choices dictated by data-availability. Consequently, our paper focuses on variations between these common measures and less on each of these approaches individually. Nevertheless, this section provides a short comparison between elite, expert, and manifesto data for the interested reader (see Table A.1.; additional reviews can be found in e.g. Mair 2001; Volkens 2007).

**Table A.1. Comparison between elite, expert and manifesto data**

|  | **Elites** | **Experts** | **Manifestos** | |
| --- | --- | --- | --- | --- |
|  |  |  | **Manual** | **Computer-assisted** |
| **Time costs** | time-consuming | time-efficient | time-consuming | time-efficient |
| **Financial costs** | cost-consuming | cost-efficient | cost-consuming | cost-efficient |
| **Time horizon** | current party positions | current party positions | current and past party positions | |
| **External dependence** | politicians | political scientists | none | |
| **Strategic nature of data source** | less strategic | non-strategic | strategic | |

First, let us look at elite data derived from surveys among politicians such as the IntUne data which ask parliamentarians to locate themselves on a given policy continuum (Cotta et al. 2007, 2009; Best et al. 2012). The implementation of elite surveys is very time-consuming and requires considerable financial resources, in particular when analyzing several countries, and the success of the data collection effort hinges on the support by the political elite addressed in the study. Successful elite surveys, however, give us the sum of the office-holders’ perspectives on their party’s position on the surveyed dimension(s). The data presents *current* party positions. They are largely detached from former party positions and their ideological ‘baggage’ and are thus particularly well suited to track changes over time. Since the elites fill out the questionnaires spontaneously and anonymously, it is reasonable to expect the data to be less influenced by strategic behavior than, for example, manifesto contents. Finally, as elites can be asked directly about how to place their party on the relevant scale, no assumptions need to be made about how specific party positions turn into party placement. However, elite surveys typically rely on samples and while their representativeness can be checked on structural factors, assumptions need to be made about the attitudinal homogeneity of the universe.

Turning now to expert data, which are, again, party position estimates based on surveys. This time, however, not parliamentarians are questioned about their ideological perspective, but country experts. Typically, political scientists specialized in a specific country provide information on party locations on relevant dimensions. The Chapel Hill Expert Survey (CHES; Hooghe et al. 2010; Bakker et al. 2015) is a well-known and widely used example of this approach. The great advantage of expert data is that they have proven to be the fastest feasible approach, even in comparative design (Bakker et al. 2015; Benoit and Laver 2006; Hooghe et al. 2010; Rohrschneider and Whitefield 2007, 2016). Obviously, this approach relies on the support by other political scientists who, luckily, seem to constitute a willing-to-respond-population. In contrast to estimates derived from political elites, however, experts are likely to resort to parties’ past behavior and their ideological rooting. Asking experts to pinpoint the *current* location of a given party in a given party system may thus be a highly demanding task that requires them to abstract from these strong queues and heuristics. Accordingly, it seems almost impossible to ask the experts to reliably position parties’ over time. This would challenge the experts’ memories and most likely lead to biased results. Lastly, one key advantage of expert data is that respondents are not strategic in a sense that experts would aim to present ‘their’ party in a most favorable light. Yet experts’ professional neutrality might be undermined if they display overwhelming sympathy for one side of the political spectrum (Curini 2010).

Let us now consider the nature of manifesto data. When it comes to turning text into data, scholars rely on either manual (Budge et al. 1987, 2001; Dolezal et al. 2016; Klingemann et al. 2006; Volkens et al. 2015) or computer-assisted coding (Laver et al. 2003; Laver and Garry 2000; Slapin and Proksch 2008). Coding data manually takes time, but benefits from human coders that are able to consider the relevant context. Computer-assisted coding, on the other hand, usually allows handling larger amount of text with fewer resources. While it carries out its routines with ultimate precision (maximizing reliability) it has limitations in dealing with, for example, implicit meanings or ambiguity (what might affect its validity; for a comparison between manually and computer assisted coding, see Pennings 2011). Similar to estimates derived from elite surveys, it is possible to analyze *current* party positions. Given that most parties publish a manifesto before each election, these documents provide the information needed to extract party positions in the past. In contrast to expert data, estimates derived from party manifestos carry a strong strategic element. Specifically, political parties drafting manifestos must master the difficult balancing act of appealing to their core electorate while appeasing often-conflicting intra-party interests in the context of prevalent ideological constraints (Grofman 1985).

**B. Literature review**

We conduct the literature review in tabular form. Table A2. is devoted to cross-validations of left–right measures while Table A.3 focuses on cross-validation research regarding the European integration dimension.

|  | **Elite survey** | **Expert survey** | **Mass survey** | **Party Manifesto analyses** |
| --- | --- | --- | --- | --- |
| **Bakker et al. 2015** | No comparison made | Bakker et al. (2015, 2010 Chapel Hill expert survey) | European Election Study (2009, EES) | Budge et al. (2001, CMP);  Volkens et al. (2006, 2010, CMP) |
|  |  | Data collection in 2011 in 28 Eastern and Western European countries | Data collection in 2009 and data from 104 parties in Eastern and Western European countries | Data from most recent election (time period 2000 to 2010) in Eastern and Western European countries |
| **Benoit & Laver 2007** | No comparison made | Benoit and Laver (2006) | No comparison made | Budge et al. (2001, CMP) |
|  |  | Data collection in 2002–2003 in 47 countries |  | Data from most recent election |
| **Keman 2007** | No comparison made | Castles & Mair (1984); Huber & Inglehart (1995); Marks & Steenbergern (1999) | No comparison made | Budge et al. (2001, CMP) |
|  |  | Data collection in 1982/83, 1993, 1999 in 18 Western countries |  | Data collection in 1981–1998 in 18 Western countries |
| **Whitefield et al. 2007** | No comparison made | Hooghe et al. (2010); Rohrschneider & Whitefield expert survey (RW) | No comparison made | No comparison made |
|  |  | Data collection from 2002 to 2004 in 9 CEE countries |  |  |
| **McDonald et al. 2007** | No comparison made | Castles & Mair (1984); Laver & Hunt (1992); Huber & Inglehart (1995) | No comparison made | Budge et al. (2001, CMP) |
|  |  | Data collection in 1982/83, 1989, 1993 in 17 Western countries |  | Average data from 1972–1998 period in 17 Western countries |
| **McDonald & Mendes 2001** | No comparison made | Castles & Mair (1984); Laver & Hunt (1992); Huber & Inglehart (1995) | No comparison made | Budge et al. (1987, CMP) |
|  |  | Data collection in 1982/83, 1989 and 1993 in 16 Western democracies |  | Average data from the 1972–1992 period in 16 countries |

**Table A.2. Literature review: Cross-validation on left–right dimension**

*Notes: Partly based on Marks (2007: 8). CMP=Comparative Manifesto Project*

|  | **Elite survey** | **Expert survey** | **Mass survey** | **Party Manifesto data** |
| --- | --- | --- | --- | --- |
| **Bakker et al. 2015** |  | Bakker et al. (2015, 2010 Chapel Hill expert survey) | European Election Study (2009, EES) | Budge et al. (2001, CMP);  Volkens et al. (2006, 2010, CMP) |
|  |  | Data collection in 2011 in 28 Eastern and Western European countries | Data collection in 2009 and data from 104 parties in Eastern and Western European countries | Data from most recent election (time period 2000 to 2010) in Eastern and Western European countries |
| **Hooghe et al. 2010** | No comparison made | Hooghe et al. (2010); 2003 Benoit-Laver expert survey; 2002 RW expert survey | No comparison made | Budge et al. (2001, CMP) |
|  |  | Subset of the data collection in 2003 in 23 Western European countries European integration |  | Data for elections in 2002 or earlier |
| **Marks et al. 2007** | Katz et al. (1999) | Steenbergen & Marks (2007) | Eijk et al. (2002) | Budge et al. (2001, EU manifesto) |
|  | Data collection in 1996 in 12 EU-countries (question on European currency) | Data collection in 1999 in 12 EU-countries (question on European integration) | Data collection in 1999 in 15 EU-countries (question on further European integration) | Data collection in 1998 (or earlier) in 12 EU countries |
| **Ray 2007** | No comparison made | Ray (1999) | EU Commission (1988, EB) | Budge et al. (1992, CMP) |
|  |  | Data collection in 1988 in 15 EU-countries | Data collection in 1988 in 15 EU-countries | Data collection in 1988 (or earlier) |

**Table A.3. Literature review: Cross-validation on European integration dimension**

*Notes: Partly based on Marks (2007:8). CMP=Comparative Manifesto Project; EB=Eurobarometer; RW=Rohrschneider & Whitefield expert survey*

**C. Information on data sources**

**Left–right IntUne 2007 and 2009:**

In politics people sometimes talk of ‘left’ and ‘right’. Where would you place yourself on a scale from 0 to 10 where ‘0’ means ‘the left’ and ‘10’ means ‘the right’, and ‘5’ means ‘neither left nor right’?

**EU IntUne 2007 and 2009:**

Some say European unification has already gone too far. Others say it should be strengthened. What is your opinion? Please indicate your views using a 10-point-scale. On this scale, ‘0’ means unification ‘has already gone too far’ and ‘10’ means it ‘should be strengthened’. What number on this scale best describes your position?

**Left–right Chapel Hill expert survey 2006 and 2010:**

Please tick the box that best describes each party's overall ideology on a scale ranging from 0 (extreme left) to 10 (extreme right).

**EU Chapel Hill expert survey 2006 and 2010:**

How would you describe the general position on European integration that the party leadership took over the course of 2006/2010?

**Operationalization of control variables**

The electoral size of political parties is captured in terms of their vote share in percent in the most recent national election prior to 2006 and 2010, respectively. A second variable indicates whether a party was in government or in opposition during the fieldwork. Party age is captured via the number of democratic elections they participated in since 1945. Even though this operationalization might be prone to bias as the number of democratic national elections for a given time period varies across countries, one might argue that it is precisely the number of active campaigns rather than the period of sole existence that determines parties’ visibility, and thus position estimates’ validity.

The party-specific salience of the general left–right dimension is obtained from the MARPOR/CMP data. We define salience simply as the share of quasi-sentences assignable to any of the 26 MARPOR/CMP left–right categories. In contrast, the relevance of the European integration project to each party is assessed based upon a CHES survey question. Therein, experts were asked to evaluate the relative salience of European integration in a party's public stance on a scale ranging from 1 (no importance) to 4 (great importance). Although using the MARPOR/CMP data might be considered a viable alternative (despite our concerns raised above), the empirical results by Netjes and Binnema (2007, 47) indicate that this approach would lead to an endogeneity problem as EU salience is itself correlated to several of our predictor variables.

All parties belonging to Eastern European countries are captured via a simple dummy variable. The expert count per party is the number of experts who evaluated the party at hand. Hence, it corresponds not to the total number of returned questionnaires per country but to the number of actual policy position estimates per party.

**Table A.4. Quadripartite data structure (N=131 parties)**

| Countries | Number of parties | Elite data | | Expert data | | Manifesto data | |
| --- | --- | --- | --- | --- | --- | --- | --- |
|  |  | Mean | Median | Mean | Median | Most recent parliamentary elections | |
| Austria | 10 | 13.2 | 11 | 11.0 | 11 | Oct. 1, 2006 | Sep. 28, 2008 |
| Belgium | 15 | 8.9 | 9 | 12.2 | 10 | May 18, 2003 | Jun. 10, 2007 |
| Bulgaria | 10 | 14.3 | 11 | 14.0 | 14 | Jun. 25, 2005 | Jul. 5, 2009 |
| Czech Republic | 8 | 15.1 | 13 | 14.1 | 14.5 | Jun. 3, 2006 | May 29, 2010 |
| Denmark | 8 | 11.5 | 10.5 | 9.8 | 9 | Feb. 8, 2005 | Nov. 13, 2007 |
| Estonia | 5 | 11.4 | 12 | 14.0 | 14 | Mar 4, 2007 | - |
| France | 5 | 26.4 | 24 | 9.0 | 9 | Jun. 9, 2002 | Jun. 10, 2007 |
| Germany | 9 | 17.3 | 11 | 18.1 | 18 | Sep. 18, 2005 | Sep. 27, 2009 |
| Greece | 2 | 43.0 | 43 | 10.0 | 10 | Mar. 7, 2004 | Sep. 16 2007 |
| Hungary | 6 | 22.8 | 22 | 11.5 | 11.5 | Apr. 9, 2006 | Apr. 11, 2010 |
| Italy | 8 | 18.8 | 20.5 | 9.5 | 9 | Apr. 10, 2006 | Apr. 13, 2008 |
| Lithuania | 8 | 10.6 | 10.5 | 9.3 | 8 | Oct. 10, 2004 | Oct. 12, 2008 |
| Poland | 9 | 16.4 | 11 | 11.1 | 8 | Sep. 25, 2005 | Oct. 21, 2007 |
| Portugal | 5 | 26.0 | 26 | 8.4 | 10 | Feb. 20, 2005 | Sep. 27, 2009 |
| Slovakia | 10 | 13.1 | 12 | 14.4 | 14 | Jun. 17, 2006 | Jun. 12, 2010 |
| Spain | 7 | 22.1 | 31 | 12.6 | 13 | Mar. 14, 2004 | Mar. 9, 2008 |
| UK | 6 | 20.2 | 17 | 13.0 | 13 | May 5, 2005 | May 6, 2010 |
| Average |  | 16.1 | 13 | 12.2 | 12 |  |  |

*Notes:* European Parliament elections were held on June 10–13, 2004 and June 4–7, 2009. No second wave elite data are available for Estonia.

*Sources:* Bakker et al., 2012; Cotta et al., 2007, 2009; Hooghe et al., 2010; Volkens et al., 2015.

Table A4 lists the countries in our sample along with the number of parties analysed in that country. In addition, it displays the average and the median number of elites and experts available by party. The average number of MPs across countries interviewed for each party is approximately 16. However, the substantial differences between mean and median number of MPs in several countries, such as Poland and Spain, indicate that elites often cluster around two or three more popular parties. While low response rates are an issue often brought forward by critics of elite survey data, the empirical evidence suggests that they do not per se directly result in significant representational bias (Fisher and Herrick, 2013; Laver, 2014). Yet, in order to increase the robustness of our empirical results we exclude any party which features less than 5 MPs or less than half of its MPs in the national legislature (to avoid excluding small parties, which hold less than 5 seats in the national legislature). With regard to the number of experts, no less than five experts evaluate the parties in our reduced sample. On average, each party in this reduced sample is assessed by twelve experts. The final columns of Table A3 show the date of the national elections from which the party manifestos were retrieved.

**Table A.5. Summary of empirical findings**

|  | Left–right | | European integration | |
| --- | --- | --- | --- | --- |
|  | Experts | Manifestos | Experts | Euromanifestos |
|  |  |  |  |  |
| **Manipulable characteristics** |  |  |  |  |
| Number of experts | n.s. $(-$) |  | n.s. $(-$) |  |
| Expert agreement | $- (-$) |  | $- (-)$ |  |
| Percent of MPs | $+$ $(-$) | n.s. $(-)$ | n.s. $(-$) | n.s. ($-)$ |
| Type of document (party-specific) |  | $- (-$) |  | n.s. $(-$) |
| Length of document |  | n.s. $(-$) |  | n.s. ($-)$ |
|  |  |  |  |  |
| **Characteristics beyond control** |  |  |  |  |
| Intra-party heterogeneity | n.s. ($+)$ | n.s. ($+)$ | $+$($+)$ | n.s. ($+)$ |
| Party position shift | $- (+)$ |  | n.s. $(+$) |  |
| Party extremism | n.s. $(+/-)$ | $+$ ($+)$ | $+(+/-)$ | $+$($+)$ |
|  |  |  |  |  |
| **Controls** |  |  |  |  |
| Party size | n.s. ($-)$ | n.s. ($-)$ | n.s. ($-)$ | $- (-)$ |
| Government party | n.s. ($-)$ | n.s. ($-)$ | n.s. ($-)$ | n.s. ($-)$ |
| Party age | n.s. ($-)$ | n.s. ($-)$ | n.s. ($-)$ | n.s. ($-)$ |
| Issue salience | n.s. ($-)$ |  | n.s. ($-)$ |  |
| Central and Eastern Europe | +($+)$ | n.s. ($+)$ | n.s. ($+)$ | n.s. ($+)$ |

*Notes*: + denotes factors that increase systematic differences between measures, - denotes factors that decrease systematic differences between measures; n.s. not significant results; ( ) not applicable; expected effects in parentheses.

# References

Bakker, R, de Vries, C, Edwards, E, Hooghe, L, Jolly, S, Marks, G, Polk, J, Rovny, J, Steenbergen, M and Vachudova, M (2015) Measuring Party Positions in Europe: The Chapel Hill Expert Survey Trend File, 1999–2010. *Party Politics* 21(1):143–152.

Bakker, R, Jolly, S, Polk, J and Poole, K (2014) The European Common Space: Extending the Use of Anchoring Vignettes. *Journal of Politics* 76(4):1089–1101.

Benoit, K and Laver, M (2006) *Party Policy in Modern Democracies*. London: Routledge.

Benoit, K and Laver, M (2007) Estimating Party Policy Positions: Comparing Expert Surveys and Hand Coded Content analysis. *Electoral Studies* 26(1): 90–107.

Best, H, Lengyel, G and Verzichelli, L (eds.) (2012) *The Europe of Elites. A Study into the Europeanness of Europe’s Economic and Political Elites*. Oxford: Oxford University Press.

Budge, I, Klingemann, H-D, Volkens, A, Bara, J, and Tanenbaum, E (eds.) (2001) *Mapping Policy Preferences: Estimates for Parties, Electors and Governments 1945–1998*. Oxford: Oxford University Press.

Budge, I, Robertson, D, Hearl, D and Volkens, A (1992) *Manifesto Research Project [computer file]*. Colchester: ESRC Data Archive.

Budge, I, Robertson, D, Hearl, DJ (eds.) (1987) *Ideology, Strategy and Party Change: Spatial Analyses of Post-War Election Programmes in 19 Democracies.* Cambridge: Cambridge University Press.

Castles, F and Mair, P (1984) Left–right Political Scales: Some “Expert” Judgments. *Europan Journal of Political Research* 12(1):73–88.

Cotta, M, Isernia, P, and Bellucci, P (2007) *IntUne Mass Survey Wave 1. ICPSR34421-v1*. Ann Arbor: Inter-university Consortium for Political and Social Research.

Cotta, M, Isernia, P, and Bellucci, P (2009) *IntUne Mass Survey Wave 2. ICPSR34272-v2*. Ann Arbor: Inter-university Consortium for Political and Social Research.

Curini, L (2010) Experts’ Political Preferences and their Impact on Ideological Bias. *Party Politics* 16(3):299–321.

Dolezal, M, Ennser-Jedenastik, L, Müller, WC, Winkler, AK (2016) Analyzing Manifestos in their Electoral Context: A New Approach Applied to Austria, 2002–2008. *Political Science Research and Methods* 4(3):641–650.

Eijk, C van der, Franklin, M, Schoenbach, K, Schmitt, H, Semetko, H, Brug, W van der, Holmberg, S, Mannheimer, R, Marsh, M, Thomassen, J and Wessels, B (2002) *European Election Study – 1999*. Hamburg: IPSOS.

Fisher, SH and Herrick, R (2013) Old versus New: The Comparative Efficiency of Mail and Internet Surveys of State Legislators. *State Politics & Policy Quarterly* 13(2):147–163.

Grofman, B (1985) The Neglected Role of the Status Quo in Models of Issue Voting. *The Journal of Politics* 47(1): 230–37.

Hooghe, L, Bakker, R, Brigevich, A, de Vries, C, Edwards, E, Marks, G, Rovny, J and Steenbergen, M (2010) Reliability and Validity of the 2002 and 2006 Chapel Hill Expert Surveys on Party Positioning. *European Journal of Political Research* 49(5): 687–703.

Huber, J and Inglehart, R (1995) Expert Interpretations of Party Space and Party Locations in 42 Societies. *Party Politics* 1(1):73–111.

Katz, R, Norris, P, Thomassen, J, and Wessels, B (1999) *The 1996 Political Representation in Europe Survey of Members of 11 National Parliaments and Members of European Parliaments*. Available from:http://www.gesis.org/ZUMA/.

Klingemann, H-D, Volkens, A, Bara, J, Budge, I, McDonald, M (eds.) (2006) *Mapping Policy Preferences II: Estimates for Parties, Electors and Governments in Eastern Europe, the European Union and the OECD, 1990–2003*. Oxflord: Oxford University Press.

Laver, M (2014) Measuring policy positions in political space. *Annual Review of Political Science* 17: 207–223.

Laver, M, and Garry, J (2000) Estimating Policy Positions from Political Texts. *American Journal of Political Science* 44(3):619–34.

Laver, M, Benoit, K, Garry, J (2003) Extracting Policy Positions from Political Texts Using Words as Data. *American Political Science Review* 97(2):311–331.

Mair, P (2001) Searching for the Positions of Political Actors. A Review of the Approaches and a Critical Evaluation of Expert Surveys. In: Laver, M (ed) *Estimating the Policy Positions of Political Actors*. London: Routledge, pp. 10–30.

Marks, G (2007) . Introduction: Triangulation and the Square-root Law. *Electoral Studies* 26(1):1–10.

Marks, G, Hooghe, L, Steenbergen, MR and Bakker, R (2007) Crossvalidating Data on Party Positioning on European Integration. *Electoral Studies* 26(1):23–38

McDonald, MD and Mendes, SM (2001) The Policy Space of Party Manifestos. In: Laver, M (ed) *Estimating the Policy Positions of Political Actors*. London: Routledge, pp. 10–30.

McDonald, MD, Mendes, SM and Kim, M (2007) Cross-temporal and Cross-national Comparisons of Party Left–Right Positions. *Electoral Studies* 26(1): 62–75.

Netjes, CE and Binnema HA (2007) The Salience of the European Integration Issue: Three Data Sources Compared. *Electoral Studies* 26(1):39–49.

Pennings, P (2011) Assessing the ‘Gold Standard’ of Party Policy Placement: Is Computurized Replication Possible? *Electoral Studies* 30(3):561–570

Ray, L (1999) Measuring Party Positions on European Integration: Results from an Expert Survey. *European Journal of Political Research* 36(2):283–306.

Ray, L (2007) Validity of Measured Party Positions on European Integration: Assumptions, Approaches, and a Comparison of Alternative Measures. *Electoral Studies* 26(1):11–22.

Rohrschneider, R and Whitefield, S (2007) Representation in New Democracies: Party Stances on European Integration in Post-Communist Eastern Europe. *Journal of Politics* 69(4):1133–1146.

Rohrschneider, R and Whitefield, S (2016) Responding to growing European Union-skepticism? The Stances of Political Parties Toward European Integration in Western and Eastern Europe Following the Financial Crisis. *European Union Politics* 17(1):138–161.

Slapin, JB and Proksch, J-O (2008) A Scaling Model for Estimating Time-Series Party Positions from Texts. *American Journal of Political Science* 52(3):705–722.

Steenbergen, MR and Marks, G (2007) Evaluating Expert Judgments. *European Journal of Political Research* 46(3):347–366.

Volkens, A (2007) Strengths and Weaknesses of Approaches to Measuring Policy Positions of Parties. *Electoral Studies* 26(1):108–120.

Volkens, A, Klingemann, H, Bara, J, Budge, I and McDonald, M (2006) *Mapping Policy Preferences II. Estimates for Parties,Electors, and Governments in Eastern Europe, European Unionand OECD 1990–2003*. New York: Oxford University Press.

Volkens, A, Lacewell, O, Regel, S, Schultze, H and Werner, A (2010) *The Manifesto Data Collection. Manifesto Project(MRG/CMP/MARPOR)*. Available at: http://manifestopro-ject.wzb.eu/.

Volkens, A, Lehmann, P, Matthieß, T, Merz, N, Regel, S and Werner, A (2015) *The Manifesto Data Collection. Manifesto Project (MRG/CMP/MARPOR). Version 2015a*. Berlin: Wissenschaftszentrum Berlin für Sozialforschung (WZB).
